# Editorial: Evidence synthesis for accelerated learning on climate solutions

**DOI:** 10.1002/cl2.1128

**Published:** 2020-12-28

**Authors:** Lea Berrang‐Ford, Friederike Döbbe, Ruth Garside, Neal Haddaway, William F. Lamb, Jan C. Minx, Wolfgang Viechtbauer, Vivian Welch, Howard White

**Affiliations:** ^1^ Priestley International Centre for Climate University of Leeds Leeds UK; ^2^ Stockholm School of Economics Stockholm Sweden; ^3^ University of Exeter Truro UK; ^4^ Mercator Research Institute on Global Commons and Climate Change Berlin Germany; ^5^ Maastricht University Maastricht The Netherlands; ^6^ Bruyère Research Institute Ottawa Canada; ^7^ Campbell Collaboration USA

By signing the Paris Agreement with the aim to limit global warming to well below 2°C relative to preindustrial levels, countries have committed to kickstart the age of climate solutions. This challenge should not be underestimated: It requires turning around a 270‐year‐old trajectory of CO_2_ emissions growth that started with the industrial revolution (Friedlingstein et al., 2019), and racing towards net zero emissions over the next 3–5 decades (IPCC, 2018). But that is not enough. Because anthropogenic carbon emissions have already caused consequential warming of about 1°C since preindustrial times, there is a further need to reduce vulnerabilities and adapt to climate impacts that cannot be avoided (IPCC, 2014, 2018). All parts of society and the economy will need to play their parts in the transformation towards a climate‐resilient, net‐zero emissions world. It requires nothing less than transformational policies at all levels of governance from local to national and international (IPCC 2014, 2018). Climate policies for mitigation and adaptation have to become the focus in science and policy if we are to have the slightest chance of living up to this challenge.

## 1 ASSESSING EVIDENCE ON CLIMATE CHANGE

Because there is no time left for trial and error and since resources for organising a transformation into a carbon‐neutral world are inherently limited, decision‐making on climate solutions needs to be based on the best available evidence. “Best” evidence should be rigorous, transparent, timely, efficient, and fit‐for‐purpose applying formal methods for evidence synthesis (Gough et al., 2017; Haddaway et al., 2020).

Knowledge brokering (Pielke, 2007) on climate change is commonly undertaken through scientific assessments of the available scientific literature. Most prominently these assessments are undertaken by the Intergovernmental Panel on Climate Change (IPCC). Like many other global environmental assessments, IPCC assessments are diverse, large‐scale, multistakeholder and intergovernmental processes (Kowarsch et al., 2017). Compared with systematic reviews and evidence (gap) maps (Saran & White, 2018), their scope is vast. IPCC assessments usually require the consideration of more or less the entire recent scientific literature on climate change that has emerged during an assessment cycle—only somewhat constrained by a government‐approved outline that provides some thematic focus.

**Figure 1:**
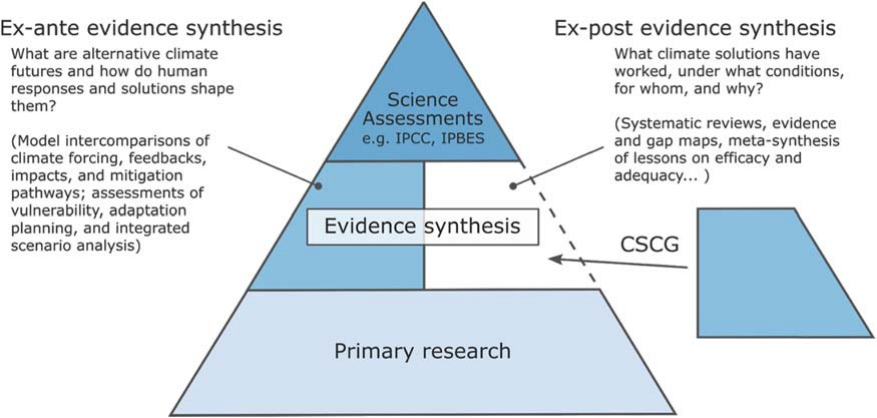
Fixing the evidence pyramid to accelerate learning on climate solutions. The systematic assessment of alternative futures via structured model intercomparisons as well as historic policy successes and failures via systematic reviews as the two foundations for comprehensive evidence synthesis on climate solutions. The CSCG aims to foster systematic review work on climate solutions. This will contribute to filling the current void in the evidence pyramid and support science assessments by the IPCC or the IPBES. CSCG, Campbell Climate Solutions Coordinating Group; IPBES, Intergovernmental Science‐Policy Platform on Biodiversity and Ecosystem Services; IPCC, Intergovernmental Panel on Climate Change

Progress in IPCC assessments depends on a well‐working evidence pyramid (Figure 1). There is no time within IPCC processes to systematically assess the vast amounts of primary research on climate change. The availability of rigorous evidence syntheses that assess the primary evidence is fundamental for learning (Berrang‐Ford et al., 2015; Minx et al., 2017).

The IPCC has made continuous progress in building up knowledge on forward‐looking questions that emerge around understanding, dealing with and mitigating potentially severe, pervasive and irreversible climate impacts that could affect human and natural systems in the absence of an adequate human response. This knowledge has had a big impact on international climate policy. It is difficult to think of major milestones like the Kyoto Protocol or the Paris Agreement being achieved without the synthetic scientific inputs provided by the IPCC. Learning has been driven by structured, model‐intercomparison exercises that provide the major backbone of the evidence synthesis tradition in climate change. Similar to systematic reviews, model intercomparison exercises set out clear protocols for systematically assessing evidence generated by ensembles of harmonised climate models on specific questions around climate science, impacts and vulnerability as well as mitigation and adaptation (Edwards, 2011; Eyring et al., 2016; O'Neill et al., 2016). These lines of synthetic evidence arising from model intercomparison exercises have been fundamental for progress in the science of climate change as well as IPCC assessment reports (Minx et al., 2019).

However, progress on understanding climate solutions from IPCC assessments has been limited, despite climate solutions now being rolled out across the world, with varying degrees of ambition and success (Eskander & Fankhauser, 2020; Höhne et al., 2020). The root of the problem is a lack of rigorous evidence syntheses that assess progress and historical success and failures in the implementation of climate solutions (Berrang‐Ford et al., 2015; Minx et al., 2017) and are designed to minimise the various sources of bias traditional literature reviews are commonly exposed to (Haddaway et al., 2020). Transparent evidence synthesis is particularly infrequent in climate research using qualitative research, largely due to the widespread perception in many social sciences that systematic approaches are highly positivist and lack the methodological flexibility needed for critical inductive inquiry. Yet qualitative research is critical for understanding the human dimensions of climate change, and we will need robust synthetic insights from the social sciences to comprehensively learn about climate solutions.

Substantial work has already been undertaken to develop qualitative evidence synthesis and mixed methods synthesis methods in other topic areas dealing with complexity. There is an opportunity to adopt and adapt these systematic evidence synthesis approaches to bridge some of the epistemological concerns. Despite this, many reviews in this field frequently lack transparent methodology about their processes, and often withdraw from engaging with the broader need for generalizeable insight (Gough et al., 2017). Systematic review research, meanwhile, has struggled to respond to the scope and heterogeneity of the climate literature, in particular the central role of contextual and mediating factors in influencing climate solutions. Thus, a robust middle layer of the evidence pyramid is partially lacking. As shown in Figure 1, the result is a lack of learning in science and policy regarding what climate solutions work under what conditions, for whom, and why in climate change assessments and beyond (Berrang‐Ford et al., 2015; Minx et al., 2017).

## 2 INVITING SYSTEMATIC REVIEWS AND EVIDENCE (GAP) MAPS ON CLIMATE SOLUTIONS

It is of great importance to grow a rigorous evidence synthesis culture particularly in the social science of climate change. The Campbell Collaboration has therefore now established a new Climate Solutions Coordinating Group (CSCG) with the purpose of building a comprehensive evidence base on climate solutions.

We are part of a growing international, multidisciplinary network of scholars committed to prepare and disseminate high quality systematic reviews and evidence and gap maps that help us better understand climate solutions. With our work we want to support better, evidence‐based decisions by: (a) supporting rigorous synthesis of evidence on key questions; (b) further improving the quality of climate change research through critical appraisal of primary research; and (c) improving the efficiency of the scientific process through enhanced oversight of, and less duplication in, secondary research efforts. We further believe that our work strengthens the IPCC and other climate change assessments by supporting the completion of its evidence pyramid (see Figure 1).

We are particularly interested in learning about what climate solutions work well under what conditions, for whom, and why. Climate change mitigation and adaptation solutions are the end‐points of our interest, but we broadly invite contributions in the field of “climate change and society”. We believe that developing a sound understanding requires a diverse approach to evidence synthesis, including quantitative, qualitative and mixed‐methods evidence (Kastner et al., 2016). We further seek evidence synthesis efforts at all levels, including systematic reviews, evidence and gap maps, systematic reviews, qualitative meta‐synthesis, mega‐maps as well as maps of maps (Saran & White, 2018).

There are scholars in very different communities working on evidence synthesis in the field of climate. We try to represent those different communities in the governance structure of the group. The Editorial Board includes researchers with a background in IPCC or similar climate change assessments as well as scholars involved in the Campbell Collaboration, Cochrane and the Collaboration for Environmental Evidence. The methodological expertise spans from more traditional quantitative systematic reviews to qualitative evidence synthesis methods such as thematic synthesis, framework synthesis, evaluation assessment, and meta‐ethnography. Similarly, the Advisory Board comprises senior management of major global environmental assessments such as IPCC, Intergovernmental Science‐Policy Platform on Biodiversity and Ecosystem Services or the United Nations Environment Programmes' Emissions Gap Reports as well as the above mentioned Collaborations. We aim to diversify the representation of marginalised groups in both Boards as CSCG matures.

Working on a cross‐cutting topic such as climate change provides many interfaces to the work by other Campbell Coordinating Groups. For example, it will be crucial to work closely with the International Development Coordinating Group as both discourses are closely intertwined in the context of international climate policy. Similar, the work by the Education Collaborating Group is relevant as the importance of civic engagement and education for organising transformative climate solutions is increasingly appreciated. Evidence synthesis on climate solutions will also require adjusting available to and developing new methods for the specific context and multidisciplinary structure of the climate discourse requiring regular engagement and cooperation with the Methods Coordinating Group. Finally, as probably true for all Coordinating Groups across Campbell, the work of the Knowledge Translation and Implmentation Coordinating Group that focuses on effective ways of communicating evidence to different audiences is of direct relevance to our work at science‐policy interface.

Part of our work will be to build more capacity for systematic reviews in the field of climate. For that we will provide training in evidence synthesis methodologies and specifically target climate change assessment communities around the IPCC. We will enhance our reach further through the provision of a set of freely available and open training resources. We recognise that access to free training and mentoring are important barriers to building capacity for evidence‐informed decision‐making (including evidence synthesis) in resource constrained contexts like the Global South. Our aim is, therefore, to provide accessible and functional training in climate evidence synthesis that works across settings (e.g., with low bandwidth internet).

## 3 TOWARDS ENHANCED EVIDENCE SYNTHESIS

It is an exciting and important task to build a thriving (social science) community that tries to learn what climate solutions work under what conditions, for whom, and why, using rigorous systematic review and evidence (gap) map methodologies. Beyond the core remit of the group to promote rigorous evidence synthesis as an important pathway to more evidence‐informed climate policy, CSCG will further try to advance evidence synthesis methodology more generally in four priority areas.

- Promote the adaptation and application of qualitative and mixed methods evidence synthesis methodologies: The social science of climate change provides a very diverse body of evidence. Only a small part of this literature is directly on impact evaluations of climate solutions based on experimental or quasiexperimental research designs (IPCC, 2014). In part, this is because climate solutions are conditional on individual and societal behaviours, institutional settings and governance structures that are impossible to fully comprehend in solely quantitative work. Qualitative evidence synthesis is also key to understanding stakeholder experience and attitudes, the feasibility and acceptability of policy proposals, as well as unintended consequences (Macura et al., 2019). In order to comprehensively learn from the available evidence on climate solutions, it is, therefore, vital to mirror the methodological diversity in primary evidence by promoting the application of the full breadth of qualitative and mixed methods evidence synthesis methodologies (Kastner et al., 2016).
- Encourage the development and application of novel evidence synthesis technology: Climate change is not only one of the most pressing policy issues of our time, but also a very prolific area of research with tens of thousands of studies being published every year. In such an age of big literature with a vast and fast‐growing evidence base, traditional manual tools of evidence synthesis are being pushed to their limits (Callaghan et al., 2020; Minx et al., 2017; Nunez‐Mir et al., 2016). As such, the field of climate is ideally suited to explore how technology can be used to streamline individual processes in evidence synthesis. The CSCG will therefore contribute to discussions and methodologies on automation and computer‐assistance for systematic reviews and evidence and gap maps (Haddaway et al n.d.; Nakagawa et al., 2019; O'Connor et al., 2019; Tsafnat et al., 2014; Westgate et al., 2018) as well as broader global environmental assessments (Callaghan et al., 2020; Lamb et al., 2019).
- Integrating the evidence synthesis traditions of the climate and the systematic review communities: It is clear that comprehensive learning on climate solutions must be rooted in a systematic exploration of alternative future pathways as well as systematic evaluation of past policies. The CSCG brings together two evidence synthesis communities with complementary strengths in ex‐ante and ex‐post assessments of climate solutions. We are convinced that there is great scope for mutual learning and that sharing and integration of these distinct traditions will ultimately lift evidence synthesis and the resulting scientific policy advice to a higher level.
- Promote the application of formal assessment of confidence in evidence within syntheses of climate solutions: Assessment of uncertainty and confidence in evidence is a critical component of climate change assessments (Janzwood, 2020; Jonassen & Pielke, 2011; Mach et al., 2017; Petticrew & McCartney, 2017). Methods for uncertainty assessment are widely used in quantitative syntheses of climate projections, and detection and attribution of impacts. Lesser known and used are methods for formally assessing confidence in mixed‐methods and qualitative evidence, which are used in the health sciences but rarely in climate research (Colvin et al., 2015; Lewin et al., 2018; Mach & Field, 2017; van Bavel et al., 2020). Learning on climate solutions must be underpinned by awareness of the evolving quality and reliability of our evidence base (Adler & Hirsch Hadorn, 2014; Aven, 2019). The CSCG will bring together expertise in systematic confidence assessment and climate solutions research to apply and promote robust confidence assessment within evidence syntheses for climate solutions.

Climate change is one of the most pressing problems that humankind faces. We need to urgently and rapidly learn from the best available evidence on climate solutions. The CSCG warmly invites registrations and submissions to Campbell Systematic Reviews from climate experts across diverse disciplines and evidence synthesis practitioners.
